# An Exposition of the Guild Rules

**Published:** 1913-07-19

**Authors:** 


### The National Medical Guild.

### An Exposition of the Guild Rules.

This and some following articles will be devoted to an exposition of the rules of the Guild. Many of these require but little elaboration, but to some considerable attention needs to be devoted. It should be at once explained that these rules have been very carefully drawn up by counsel, and that it will probably be necessary to add to them in the future.

Before the passing of the Trade Union Act, 1913, almost any rules could be registered, and the fact that its rules were registered did not give a trade union the right, in a Court of Law, to say: "As a trade union we are exempt from such and such actions." It was required to prove that it was in fact acting as a trade union, *i.e.*, in restraint of trade or regulation of the profession of its members.

But from the date of the Royal Approval to the Trade Union Act, 1913, it became necessary that rules should be much more carefully drafted, since the mere fact of registration is now legal proof that a body is a trade union in name and deed. It is not now necessary to submit other proof to the Courts than the certificate of registration.

### Rule 1.—Name of Society and Office.

The Society (hereinafter referred to as the Guild) shall be called the National Medical Guild. Its registered office is in London, and is 34 Villiers Street, Strand, W.C., which is the place of meeting for the business of the Guild, and is the office to which all communications and notices may be addressed.

Several comments arise on this rule. Firstly, as to the name. It was not at all easy to find a suitable name. The words "British," "Medical," and "Society," were all ruled out as tending to confuse the Guild with other bodies. The words "Medical Guild" alone could not be used as there are already other medical guilds in existence. The addition of the word "National" served to distinguish the Guild from others, *e.g.*, those at Edinburgh and Belfast, and also showed that it was not intended as a local body for one part of the United Kingdom but to cover all parts.

The present registered office is only intended to be a temporary home for the Guild, but it is necessary to register some address and this was therefore given.

### Rule 2.—Objects.

This is perhaps one of the most important rules from a legal point of view, and it will be noted that its wording is of the type which is supposed to appeal with peculiar power to the legal mind, while exasperating more normal persons. It is as follows:—

The objects of the Guild are to regulate the relations between medical practitioners and their employers, and between medical practitioners and medical practitioners; to regulate the rates of payment; to regulate and control the conditions of contract practice; and to make provision for the creation of an organisation or organisations to supply medical service; to establish a fund or funds; to promote legislation; to take active part in all lawful political propaganda in the interests of the medical profession; to further the interests of the medical profession in all legitimate ways, and to employ the funds of the Guild for such purposes; to give legal assistance to members and officers; to provide for the insurance of members, either directly or by an arrangement with insurance companies, against death, illness, third party liability and/or damage caused to and by motor-cars or other vehicles; to publish books, papers, and literature in furtherance of the ends of the Guild, or in the interests of the medical profession; to federate or amalgamate with any other body or society; to acquire, lease, or build premises for the purposes of the Guild.

This rule is drawn as wide as possible in order to cover not only those activities of the Guild which are contemplated at the present time, but also any form of activity that it may be necessary to undertake in the future.

The regulation of conditions of practice means interference in the case of under-payment by the State or any body of persons, but it does not mean interference with private practice. No attempt would ever be made to lay down a scale of fees which a practitioner must accept on pain of expulsion. It is necessary to state this clearly since some members have not felt quite sure about the point.

### Public Medical Services.

It will be noted that powers are taken to "supply medical service." Such service would be in the nature of a public medical service of the sort which is very successfully run by the profession in Leicestershire, Southampton, Kent, and elsewhere. The Guild, although not attempting to lay down the proper fees for such a medical service, a matter which will be differently decided in various localities, is very willing to assist members should any trouble arise in connection with the public medical service of the locality to which they belong or, if desired, to put at their disposal details of the service which seems most likely to suit the locality in which they reside, supposing that has not yet a public service. These details vary in different parts of the country, and some are more highly developed in one respect, some in others. It is an obvious advantage to have a central body prepared to submit a scheme if desired and to advise on any legal points arising therefrom.

It should be remembered that the scale of fees in force in these public services will probably be very carefully scrutinised by the Government prior to introducing any measure for the extension of medical benefits to the dependants of the insured. It is therefore desirable that these fees should be as large as possible, consonant, of course, with the ability of the class treated to pay them without feeling that they are unjust.

### Defence Fund.

"To establish a fund or funds." In this phrase resides the right to raise a fund for the defence of the profession—a fund which shall not be open to attack on the grounds of conspiracy, etc. This is not a "trust fund" or any variety of fund which any other body can raise. It is raised in a very definite manner (which will be discussed when Rule 18 is dealt with) and is used for very definite purposes. The latter are set out in this rule. The fact that the raising of the fund and the purposes of the fund are duly set out in the objects of the Guild is all-important. Any body which should raise such a fund without having this stated in its rules would be liable to just those attacks from which a trade union is immune. It is to be regretted that the British Medical Association seems to have some bias in favour of raising a fund by other means than through a registered trade union. To do so is to court disaster so soon as ever the time comes to use this fund—disaster not only for the Association but for the whole profession. This is most clearly evidenced in every legal opinion which they have obtained and published in the *Journal* of recent dates.

Apparently the chief objection of the British Medical Association to leaving this matter to a trade union not identical with the Association is a fear that the two might eventually drift apart. There can be no grounds for this, if both represent the needs of the profession. If they do not, the loss of one of them is not a matter for regret except on sentimental grounds. In Germany the same problem has been met quite successfully by means of a trade union; and, although Germany has had an Insurance Act for twenty years, the trade union and the Association have not yet shown any signs of being unable to work together for the interests of the profession. One might almost call the relation between the two an *entente cordiale*. The enemy of one is necessarily the enemy of the other and always will be so, hence the *entente* endures. Our political *entente* with a neighbouring country might be upset by some new European complication which should be hostile to one party and not to the other, but this could not be so with the profession—which must be attacked as a whole.

### To Further the Interests of the Profession.

"To further the interests of the profession in all legitimate ways" is a comprehensive object and would cover almost any activity, except those which are forbidden to a trade union, *e.g.*, trading for profit. A trade union could not run a scientific journal and make a profit out of it, although it could take steps to acquaint its members of its proceedings by means of a publication, of which any profits did not accrue to the Guild.

### Legal Assistance for Members.

"To give legal assistance to members and officers" is self-explanatory to a large extent. It will be dealt with more fully subsequently.

### Insurance Benefits.

It will be noted that full powers are taken to insure members. At present not much has been done in this matter but a certain reduction on premiums with any company is assured to members of the Guild. The reduction (which does not hold for life policies, but does for every other sort) varies from 10 per cent. to 25 per cent. of each premium. The steps to obtain it are simple. When a member of the Guild first joins he is sent a paper by its agents, Messrs. Lyon, Lohr, and Sly, of Gracechurch Street, E.C., and on this is stated the scale of reductions to which members are entitled. He has then only to send to them his expiring policy (*e.g.*, for fire, workmen's compensation, etc.). They do the rest. In return he receives a new policy with the Royal Exchange Assurance Company, and the premium on this is from 10 to 25 per cent. cheaper every year than was the old policy. It is, therefore, quite possible for many members to save their annual subscription by reductions on their insurance policies. Members are therefore advised to take advantage of this arrangement whenever a policy payment falls due.

In the future it may be possible for the Guild to bear its own insurance risks, but that is not desirable at present, and hence an arrangement has been come to which is distinctly advantageous to the profession—or rather to such members as join the Guild.

